# Data envelopment analysis for ambulance services of different service providers in urban and rural areas in Ministry of Health Malaysia

**DOI:** 10.3389/fpubh.2022.959812

**Published:** 2023-01-06

**Authors:** Nor Zam Azihan Mohd Hassan, Mohd Shahri Bahari, Farhana Aminuddin, Mohd Shaiful Jefri Mohd Nor Sham Kunusagaran, Nur Amalina Zaimi, Ainul Nadziha Mohd Hanafiah, Fakarudin Kamarudin

**Affiliations:** ^1^Institute for Health Systems Research, National Institutes of Health, Ministry of Health Malaysia, Shah Alam, Selangor, Malaysia; ^2^School of Business and Economics, Universiti Putra Malaysia, Serdang, Selangor, Malaysia

**Keywords:** technical efficiency, DEA, ambulance, healthcare, pre-hospital

## Abstract

**Introduction:**

Ambulance services are pivotal in any country's healthcare system. An efficient ambulance service not only decreases patient mortality rate but also allows resource prioritization for better outputs. This study aims to measure the efficiency of ambulance services provided by health facilities in the Ministry of Health (MOH), Malaysia.

**Methods:**

This cross-sectional study analyzed the efficiency of 76 Decision-Making Units (DMUs) or health facilities, consisting of 62 health clinics and 14 hospitals. Data Envelopment Analysis (DEA) was used for computing efficiency scores while adopting the Variable Return to Scale (VRS) approach. The analysis was based on input orientation. The input was the cost of ambulance services, while the output for this analysis was the distance coverage (in km), the number of patients transferred, and hours of usage (in hours). Subsequent analysis was conducted to test the Overall Technical Efficiency (OTE), the Pure Technical Efficiency (PTE), the Scale Efficiency (SE), and the Return to Scale with the type of health facilities and geographical areas using a Mann-Whitney U-test and a chi-square test.

**Results:**

The mean scores of OTE, PTE, and SE were 0.508 (±0.207), 0.721 (±0.185), and 0.700 (±0.200), respectively. Approximately, 14.47% of the total health facilities were PTE. The results showed a significant difference in OTE and SE between ambulance services in hospitals and health clinics (*p* < 0.05), but no significant difference in PTE between hospitals and clinics (*p*>0.05). There was no significant difference in efficiency scores between urban and rural health facilities in terms of ambulance services except for OTE (*p* < 0.05).

**Discussion:**

The ambulance services provided in healthcare facilities in the MOH Malaysia operate at 72.1% PTE. The difference in OTE between hospitals and health clinics' ambulance services was mainly due to the operating size rather than PTE. This study will be beneficial in providing a guide to the policymakers in improving ambulance services through the readjustment of health resources and improvement in the outputs.

## Introduction

Providing better healthcare services has always been one of the challenges faced by many countries. With increasing healthcare cost, budget constraints, and increasing demands, the provision of healthcare services has been more than just an issue to behold but require actions and carefully planned strategies (1). The challenges were to prioritize the resources so that every resource invested is able to produce the optimum outputs. Thus, the country's respective health systems should aim to operate at the most efficient level. Efficiency analysis is one of the many possible approaches that are beneficial in identifying the best possible way to allocate resources. Economists had also claimed that one of the major criteria for priority setting is the achievement of (high) efficiency from the limited resources available (2).

Ambulance service is considered one of the most important and fundamental aspects of health service delivery which requires many resources. Hence, it is also subjected to similar issues such as increasing healthcare costs, budget constraints, and increasing demands. The term “ambulance” came from the Latin word “ambulare”, meaning “to walk or move about” (3). It is generally known as a type of vehicle which transports injured or sick patients to any healthcare facility to receive appropriate treatment. Nonetheless, the ambulance service has evolved over the years and currently has many pivotal roles in the provision of health services. The provision of ambulance services differs between countries by their protocols and approaches (3, 4). For example, each country has set its own set of national standards for ambulance design; subject to change over time and with the introduction of different types of vehicles to cater to different demands, scenarios, terrain, landscape, financial resources, and others (5). In some European countries, ambulance services or also known as emergency services require a physician or nurse specially trained in Advanced Life Support (ALS). On the contrary, the United States (US), the United Kingdom (UK), and Australia only require paramedics to operate the ambulance services and rarely involve physicians (6, 7).

Similarly, the provision of ambulance services in Asia differs between countries. Countries like Japan, Korea, and Singapore, which are considered more developed tend to have more mature and systematic ambulance service delivery systems or also known as Emergency Medical Services (EMS) systems compared to the less developed Asian countries (4, 8, 9). In Malaysia, the ambulance service has existed for more than a decade as a pre-hospital service care. The provision of ambulance services in Malaysia covers a range of services, mainly basic transportation (“scoop and run”), provision of basic life support and first aid, as well as advance life support (10). While the Ministry of Health (MOH) Malaysia remains the main provider of ambulance services in Malaysia, other agencies such as the St John's Ambulance of Malaysia, the Malaysian Red Crescent Society, the Civil Defence, university hospitals, and private hospitals also play an important role in ambulance service delivery in Malaysia (11). Consequently, the Malaysia Emergency Response Service 999 (MERS 999) system was established by the Government of Malaysia in 2007 for better coordination between ambulance services and EMS. The system was also incorporated with the MOH hospitals through the Medical Emergency Call Centre (MECC). The ambulance services in MOH Malaysia were not solely provided by the hospitals, but also by the health clinics with the aim of expanding the coverage of the health service (10). Therefore, MECC provides the platform for better coordination of these ambulance services between hospitals and health clinics.

In 2017, the number of ambulances amounted to 2,039 located throughout all healthcare facilities within MOH Malaysia. Out of these, 1,125 (55.17%) ambulances were in hospitals, and the remaining 914 (44.83%) were located in health clinics (12, 13). Due to the scarce health resources, some of these ambulances are already aged and lack the necessary equipment (14). Nonetheless, according to the latest statistics, the demand for ambulance services in Malaysia is increasing, supported by the increasing number of road traffic accidents. In 2019, the Royal Malaysian Police (PDRM) reported that the rate of road traffic accidents was 17.4 per 1,000 population and this trend is increasing by year (15, 16).

Notwithstanding the abovementioned facts, an efficient ambulance service is crucial in healthcare delivery systems in reducing mortality and disability (17). Efficiency can be simply defined as the attainment of outputs from the set of given inputs. Thus, ambulance services are considered efficient by achieving the maximum feasible level of output from the allocated resources (or inputs) (1). Inefficiency occurs when the ambulance services fail to produce the maximum output (18). There are at least two types of efficiency, namely, Allocative Efficiency (AE) and Technical Efficiency (TE) (19). AE refers to the production of a correct mix of outputs from a given set of inputs or vice versa, whereas TE describes the maximization of the production of outputs in relation to the inputs (20).

The two common methods of measuring the efficiency level are the non-parametric and the parametric or econometric model. Both methods computed the efficiency frontiers by measuring the relative efficiency for all the Decision-Making Units (DMUs). The parametric model employed a stochastic approach and econometric function to measure the efficiency frontier. Inefficiency is indicated when DMU falls under the frontier. The parametric model measures the random error in computing the inefficiencies. The inefficiency can be separated from the total component of random error in the data. The non-parametric frontier model also known as Data Envelopment Analysis (DEA) is based on the mathematical programming model that observes all the data to produce the production frontiers for computing the efficiency scores. It was first introduced in 1978 and has since gained much attention and is used for the measurement of efficiency scores in various sectors including the health sector (21, 22). Compared to the parametric methods, the DEA is more flexible but does not take into account the random error in the data. Similarly, DMUs that lie on the efficiency frontier are considered as most efficient, while those that fall under the line are inefficient. Both have their own advantages and disadvantages (23, 24). While some researchers prefer either one of the methods, others suggest using multiple techniques for more detailed information on the efficiency measures (25–27).

The DEA has been adopted in many studies to measure efficiency and performance beyond health. A case study on the base realignment and closure (BRAC) decision process at the U.S. Department of Defense (DoD) adopted a fuzzy DEA model to measure the efficiency of 40 military bases (28). This study addressed the complex socio-economic problem using three fuzzy DEA models to inform decision-makers about restructuring. Rather than applying DEA fuzzy model, other studies integrate multiple objective linear programming (MOLP) formulation to solve the DEA problem. For example, a study combines MOLP as well as Zionts–Wallenius's method with the CCR model to analyze the performance of 20 bank branches (29). The proposed model solves the DEA problem and identifies the most preferred solution (MPS) by applying interactive MOLP. The DEA method and the interactive MOLP also have been adopted in a pilot study to decide the ideal candidates for NATO expansion as well as to assess the performance and decide on the closure of public schools in Philadelphia (30, 31). In another study, an integrated DEA and a simulation method were used for group consensus ranking instead of the other voting exercise (32). The study found that simulation can be very useful in analyzing the ranks and converting them into one group of ranked candidates.

The MOH Malaysia has been providing ambulance services as part of the healthcare service delivery at hospitals and health clinics for decades. Despite the yearly budget allocation, there is a lack of data on the efficiency of ambulance services provided by MOH Malaysia. Hence, the aim of this study is to measure the technical efficiency of ambulance services in MOH facilities and to determine the difference in the efficiency level by the type of health facility and geographical areas. Findings from this study would assist policy planning for the improvement of ambulance services in MOH facilities.

## Materials and methods

This cross-sectional study involves a total of 76 healthcare facilities in Malaysia, of which 62 were health clinics and 14 were hospitals, selected using a stratified random sampling method. Initially, Malaysia was divided into zones, from which states were randomly selected. Subsequently, each state was divided into urban and rural areas, from which hospitals and health clinics were randomly selected. For confidentiality purposes, the names of the hospitals and health clinics were not revealed. All hospitals and health clinics were given a code each for the purpose of this study (e.g., SH01, SH02, and others). Primary and secondary data were collected from March 2019 to December 2019. Data from 239 ambulances were collected from these healthcare facilities. These data include the input and output data for the efficiency analysis. The cost of ambulance services was estimated using a mix of activity-based costing (ABC) and a top-down method approach. The cost of ambulance services includes personnel cost, ambulance cost, maintenance, and overhead cost. The cost data are valued using the Malaysian Ringgit (MYR).

### Data envelopment analysis measurement

This study employed a DEA to measure the technical efficiency of each DMU. Many studies on the health sector have opted DEA for measuring the efficiency level (33, 34). The DEA is a non-parametric and linear programming method for computing the efficiency level by constructing a frontier line over the data (23). This benchmarking or comparative method allows the model to demonstrate the most optimal DMUs. These DMUs then become the benchmark for the other less-efficient DMUs (35). This study adopted the input-orientation method and the Variable Return to Scale (VRS) approach in order to compute the Overall Technical Efficiency (OTE) and its decomposition of Pure Technical Efficiency (PTE) and Scale Efficiency (SE). The input-orientation method was preferred to allow policymakers to reduce the input, which is the cost of providing ambulance services in improving efficiency. Compared to the non-oriented model in which input and output are allowed to change simultaneously, the input-oriented approach only allows the input to be changed while letting the output constant. Since the output measures used in the analysis (i.e., distance covered, number of patients transferred, and the hours of usage) are not directly under the control of each facility, hence, the input-oriented method is more appropriate to use. The VRS approach was employed due to the fact that not all health clinics and hospitals were operating at optimal levels. An efficiency score of 1 (or 100%) indicates the most efficient level and <1 (or 100%) reflects inefficiency. The Data Envelopment Analysis Programme version 2.1 (DEAP 2.1) software designed by Coelli was used for the analysis (36).

### Constant return to scale vs. variable return to scale model

The presence of imperfect competition, regulations by the government, and financial or budget constraints result in the firms or DMUs operating at suboptimal levels (23). Hence, violating the assumption of CRS that all technical efficiencies are the results of managerial issues or inefficiencies. Subsequently, the VRS model was developed by Banker et al. to solve this issue (37). According to the VRS model, TE of CRS comprises both TE of VRS and SE. The mathematical relationship between VRS and CRS can be explained by the following equation (23):


$$\begin{array}{l} TE_{CRS}=TE_{VRS}^{*}SE \end{array}$$

#### The constant returns to scale model

Efficiency for multiple inputs and multiple outputs is often defined as the weighted sum of the outputs divided by the weighted sum of the inputs. Thus, it can be summarized as follows:


(1) $$\begin{array}{l} Efficiency=\frac{weighted\;sum\;of\;outputs}{weighted\;sum\;of\;inputs} \end{array}$$

The constant return to scale model was developed by Charnes et al., which is also known as the CCR model (21). For *n* number of DMUs, *m* number of input, and *s* number of outputs, the efficiency score of the respective DMU *p* is derived from the following model:


(2) $$\begin{array}{l} \;Efficiency_{p}=Max\frac{\sum_{r=1}^{s}U_{r}Y_{rp}}{\sum_{i=1}^{m}V_{i}X_{ip}}\\ \;s.t:\;\frac{\sum_{r=1}^{s}U_{r}Y_{rj}}{\sum_{r=1}^{m}V_{i}X_{ij}}\leq 1;j=1,2,\ldots ,n\\ U_{r},\;V_{i}>0;\forall_{r},\forall_{i};r=1,2,\ldots ,s;i=1,2,\ldots ,m \end{array}$$

where *X*_*ij*_ = the amount of input *i* utilized by the *j*^*th*^ DMU;

*Y*_*rj*_ = the amount of output *r* produced by the *j*^*th*^ DMU;

*U*_*r*_ = weight given to the output *r*;

*V*_*i*_ = weight given to input *i*;

A linear programming model is derived from the functioning model Equation (2) by incorporating a constraint below:


(3) $$\begin{array}{l} \overset{m}{\underset{i=1}{\sum }}V_{i}X_{ip}=1 \end{array}$$

Hence, the efficiency score for DMU *p* is computed through the equation below:


(4) $$\begin{array}{l} Max\;Efficiency_{p}=Max_{U_{r}V_{i}}\sum_{r=1}^{s}U_{r}Y_{rp}\\ \;s.t:\;\sum_{r=1}^{s}U_{r}Y_{rj}-\sum_{i=1}^{m}V_{i}X_{ij}\leq 0;\forall_{i}\\ \;\sum V_{i}X_{ip}\;=\;1\\ \;U_{r},V_{i}>0;\forall_{r},\forall_{i} \end{array}$$

The model takes into account two constraints. The first constraint is that all the DMUs are on or below the frontier. The second was that the weighted sum of all inputs is equal to one.

#### The variable returns to scale model

The VRS model allows the separation of the overall technical efficiency into PTE and SE. Hence, it is preferred in this study for a detailed analysis of pure management efficiency. While CRS would provide only one efficient DMU, the VRS permits multiple DMUs to become efficient since the data are closely enveloped in the model. The VRS approach is based on the model developed by Banker, Charnes, and Cooper (BCC model) (37). VRS allows inefficient DMUs to be compared with relatively efficient DMUs of relatively the same size. Hence, the efficiency score for DMU p is acquired through the equation below:


(5) $$\begin{array}{l} Max\;Efficiency_{p}=Max_{U_{i}V_{i}}\sum_{r=1}^{s}U_{r}Y_{rp}+U_{0}\\ \;s.t:\sum_{r=1}^{s}U_{r}Y_{rj}-\sum_{i=1}^{m}V_{i}X_{ij}+U_{0}\leq 0;\forall_{i}\\ \;\sum V_{i}X_{ip}\;=\;1\\ \;U_{r},V_{i}>0;\forall_{r},\forall_{i} \end{array}$$

where

*U*_0_ = the convexity constraint and its sign determine the return to scale;

*U*_0_ < 0 indicates increasing return to scale;

*U*_0_ > 0 indicates decreasing return to scale;

*U*_0_ < 0 indicates constant return to scale.

### Input and output variables for DEA

In measuring the efficiency level using DEA, a set of inputs and outputs is required. There have not been any definite criteria for the selection of inputs and outputs. Nonetheless, the number of DMUs should follow the rule of thumb as follows (37, 38):


$$\begin{array}{l} n\geq max\left\{m\times s,\;3\left(m+s\right)\right\} \end{array}$$

where

*n* = number of DMUs

*m* = number of inputs

*s* = number of outputs.

Since the number of DMUs is 76, the selection of one input and three outputs for the DEA in this study is justified. Cost of providing ambulance services per month was selected as the input for the analysis. Whereas, the outputs were the distance covered (in km), number of patients transferred, and the hours of usage (in hours) per month. Data for both input and outputs were based on the year 2019 data.

The cost of ambulance services reflects the budget provided by the ministry of health to run the ambulance services for each facility. The three outputs selected in the efficiency analysis reflect the usage and utilization of ambulance services. The higher utilization of service means better ambulance usage for the money spent on the ambulance service for the respective facility. The knowledge of utilization and its outcome are important and reflect the quality of ambulance services provided (39). The distance covered by the ambulance reflects the usage of the ambulance. Distance is often described as one of the factors affecting the performance of ambulance service. Distance or km covered by ambulance has been used in previous studies to measure the efficiency of ambulance services (40, 41). The ambulance's main role is to ensure the survival of emergency patients, hence, transporting a patient who requires emergency services at the healthcare facilities (42). This transfer can either be between facilities or from the place of the emergency event to the health facilities. The hour of usage very much depends on the transport time and the geographical terrain, which is used many times in ambulance performance studies (39). While the distance of transporting may not be far, the road conditions, geographical terrains, and traffics conditions may result in a long transport time (39). The hour of usage reflects the total transportation time, which also depends on the number of patients transferred. All these three combinations of outputs reflect the complex intersection of ambulance service utilization at the respective facilities.

### OTE, PTE, SE, and return to scale analysis

If there is a difference in the two OTE scores of DMU, it indicates that the DMU has inefficiency either from the PTE or SE score. The OTE, PTE, SE, and Return to Scale were tested against the type of facility (0 = health clinics, 1 = hospitals) and the geographical area of the facility (0 = rural, 1 = urban). The Mann–Whitney *U*-test and the chi-square test were conducted using IBM SPSS 26. A *p*-value of < 0.05 is considered statistically significant. The results would also incorporate the effect size since the statistically significant *p*-value does not necessarily reflect the practical significance as determined by the effect size (43). The effect size reflects the real magnitude of the differences, thus complementing the *p*-value. The effect size for Mann–Whitney *U*-test, *r* is measured as below:


$$\begin{array}{l} effect\;size,\;r=\frac{Z}{\sqrt{N}} \end{array}$$

Effect size, *r*, of 0.1–0.29 is interpreted as a small effect, 0.30–0.49 as a medium effect, and 0.50–1.0 as a large effect (44).

As for the chi-square test, the effect size is determined by the phi coefficient. It is a correlation coefficient that ranges from 0 to 1. Similarly, the Phi coefficient of 0.1 is interpreted as a small effect, 0.30 as a medium effect, and 0.50 as a large effect (44).

## Results

### Input and output description

Table 1 shows the description of the input and outputs of ambulance services. The mean cost of ambulance services was MYR 33,805.19 (±49,930.47). Whereas, the mean distance coverage was 6,723.85 (±9,608.51) km, the number of patients transferred was 118 (±189), and hours usage was 227.82 (±300.40) hours.

**Table 1:** Descriptive statistics of input and output variables.

**Facilities**	**Input**		**Output**	

	**Cost (MYR)**	**Distance coverage (km)**	**Number of patient transferred**	**Hours usage (h)**
SH01	144,911.47	17,242.00	294	859.87
SH02	187,037.56	29,796.25	977	1,071.71
SC01	18,841.53	2,784.25	80	222.21
SC02	9,233.35	1,877.50	58	161.96
SC03	7,367.07	455.50	39	26.37
SC04	9,822.69	1,933.00	52	159.07
SC05	6,318.89	689.25	24	33.77
SC06	6,274.02	1,094.75	29	54.44
SC07	25,557.33	4,059.50	120	328.50
SC08	10,761.49	2,818.75	85	196.73
SC09	7,858.56	241.75	171	59.92
SC10	8,055.48	516.75	17	54.31
SC11	21,293.30	6,129.00	55	175.63
SC12	11,854.74	4,073.25	122	196.00
JH01	89,281.13	16,466.25	287	500.42
JH02	203,158.92	45,158.25	688	857.00
JC01	18,731.48	5,242.50	71	201.40
JC02	13,797.32	4,806.50	37	99.50
JC03	24,537.16	2,369.50	80	221.27
JC04	13,333.95	3,144.75	41	115.88
JC05	29,342.45	7,481.75	162	392.14
JC06	11,789.04	1,339.25	57	103.35
JC07	8,427.73	993.75	80	41.73
JC08	12,003.96	3,641.25	51	110.25
JC09	29,083.05	6,757.50	96	264.25
JC10	12,880.11	2,312.00	51	134.09
KH01	95,394.12	21,791.75	194	642.17
KH02	220,191.76	44,580.75	1,032	1,851.00
KC01	7,318.69	1,571.75	22	43.38
KC02	8,384.58	931.75	63	28.47
KC03	14,457.13	3,052.50	48	128.19
KC04	8,290.01	1,023.00	17	39.50
KC05	8,347.56	630.75	38	25.80
KC06	10,619.76	2,013.25	73	165.41
KC07	4,763.16	2,056.50	21	34.05
KC08	8,091.03	702.00	10	33.80
KC09	6,762.81	967.25	19	38.28
KC10	6,306.96	1,336.75	7	5.51
KC11	13,493.89	4,140.00	80	254.58
TH01	89,341.48	15,255.75	129	592.50
TH02	128,225.04	15,460.25	328	374.56
TC01	36,876.23	10,236.75	236	526.27
TC02	9,704.38	2,873.50	21	62.50
TC03	21,486.72	4,621.75	64	253.88
TC04	5,859.90	3,917.50	53	69.73
TC05	16,677.45	6,821.00	45	90.78
TC06	10,485.65	2,373.50	57	93.56
TC07	17,551.26	4,819.25	132	294.45
TC08	10,896.42	1,128.50	58	125.75
TC09	16,111.03	5,123.50	163	346.56
TC10	12,289.67	3,159.75	64	154.69
BH01	137,393.87	15,887.25	476	410.19
BH02	80,917.70	14,306.00	163	322.53
BC01	14,325.54	1,742.25	170	140.10
BC02	9,952.72	944.75	15	57.25
BC03	9,184.73	1,795.25	37	88.54
BC04	18,442.08	3,042.25	75	176.49
BC05	5,054.99	307.50	26	3.37
BC06	8,082.99	4,363.50	45	45.25
BC07	35,808.91	14,619.75	52	128.65
BC08	3,540.33	542.25	8	18.27
BC09	13,718.82	5,912.25	44	178.56
RH01	64,306.50	19,082.50	57	405.74
RH02	16,241.04	1,447.50	5	50.44
RH03	163,969.03	40,809.50	122	1,106.07
RH04	125,427.28	19,937.00	539	438.63
RC01	12,893.69	5,359.75	24	63.00
RC02	20,960.59	6,282.50	33	157.88
RC03	10,527.85	1,927.00	20	63.88
RC04	15,420.88	2,302.75	53	154.25
RC05	9,075.02	245.50	27	18.00
RC06	14,900.36	684.25	13	37.56
RC07	16,838.60	7,567.00	43	71.69
RC08	20,085.23	5,669.00	49	194.88
RC09	5,098.00	1,103.75	27	25.69
RC10	7,547.33	1,117.5	23	40.70
Minimum	3,540.33	241.75	5	3.37
Median	13,413.92	3,047.38	54	131.37
Mean	33,805.19	6,723.85	118	227.82
Maximum	220,191.80	45,158.25	1,032	1,851.00
Standard deviation	49,930.47	9,608.51	189	300.40

### Technical efficiency of ambulances services

**Figure 1:**
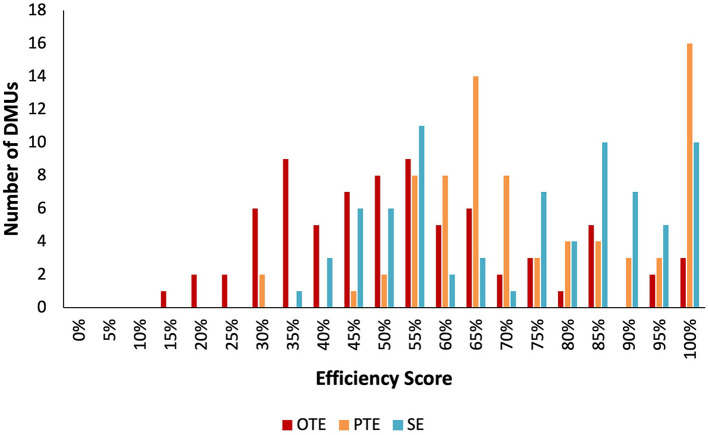
Overall technical efficiency, pure technical efficiency, and scale efficiency scores distribution.

The DEA efficiency score of ambulance services for 76 hospitals and health clinics (or DMUs) is shown in Table 2. Overall, 3.95% of the DMUs were constant to scale technical efficiency (or OTE). Around 14.47% were the variable return to scale technical efficient (PTE) and 3.95% of the DMUs were scale efficient. The mean values for OTE, PTE, and SE were 0.508 (±0.207), 0.721 (±0.185), and 0.700 (±0.200), respectively. The minimum value for OTE was 0.125 whereas the lowest values for PTE and SE were 0.287 and 0.310, respectively. From the total 73 DMUs that were not constant return to scale technically efficient (or not OTE), 60.27% were categorized as increasing return to scale (irs) and the remaining 39.73% were decreasing return to scale (drs). Of all the DMUs, health clinic BC08 was benchmarked 43 times and hence was the most variable return to scale technical efficient (PTE) score. Figure 1 shows the frequency distribution of the OTE, PTE, and SE scores.

**Table 2:** DEA efficiency score of ambulance services for hospitals and clinics (*n* = 76).

**DMUs**	**Technical efficiency**			**Return to scale**	**Rank**	**Peers^a^**

	**OTE**	**PTE**	**SE**			
**Hospitals**						
SH01	0.304	0.573	0.531	drs	60	0
SH02	0.411	1	0.411	drs	10	1
JH01	0.353	0.696	0.507	drs	34	0
JH02	0.352	1	0.352	drs	11	0
KH01	0.430	0.854	0.503	drs	22	0
KH02	0.469	1	0.469	drs	6	8
TH01	0.374	0.633	0.591	drs	46	0
TH02	0.231	0.486	0.475	drs	72	0
BH01	0.264	0.604	0.436	drs	53	0
BH02	0.292	0.547	0.534	drs	64	0
RH01	0.478	0.965	0.495	drs	13	0
RH02	0.184	0.298	0.617	irs	75	0
RH03	0.449	1	0.449	drs	9	3
RH04	0.334	0.798	0.418	drs	27	0
**Health clinics**						
SC01	0.548	0.602	0.910	irs	55	0
SC02	0.815	0.979	0.833	irs	12	0
SC03	0.311	0.593	0.524	irs	56	0
SC04	0.753	0.909	0.828	irs	19	0
SC05	0.317	0.678	0.467	irs	37	0
SC06	0.454	0.796	0.570	irs	28	0
SC07	0.598	0.603	0.990	irs	54	0
SC08	0.850	0.964	0.882	irs	14	0
SC09	1	1	1	–	5	12
SC10	0.313	0.611	0.513	irs	51	0
SC11	0.534	0.670	0.796	drs	40	0
SC12	0.928	0.945	0.983	drs	17	0
JC01	0.610	0.674	0.904	drs	38	0
JC02	0.555	0.647	0.857	drs	43	0
JC03	0.419	0.461	0.909	irs	73	0
JC04	0.501	0.558	0.898	irs	63	0
JC05	0.673	0.832	0.809	drs	24	0
JC06	0.433	0.589	0.735	irs	58	0
JC07	0.534	0.669	0.798	irs	41	0
JC08	0.579	0.608	0.952	irs	52	0
JC09	0.511	0.629	0.813	drs	47	0
JC10	0.507	0.621	0.817	irs	49	0
KC01	0.391	0.627	0.623	irs	48	0
KC02	0.435	0.615	0.707	irs	50	0
KC03	0.485	0.545	0.890	irs	65	0
KC04	0.270	0.528	0.510	irs	66	0
KC05	0.282	0.525	0.537	irs	67	0
KC06	0.724	0.864	0.838	irs	21	0
KC07	0.646	0.962	0.672	irs	15	0
KC08	0.217	0.511	0.424	irs	70	0
KC09	0.317	0.641	0.495	irs	44	0
KC10	0.317	0.648	0.489	irs	42	0
KC11	0.902	0.936	0.964	irs	18	0
TC01	0.724	1	0.724	drs	7	8
TC02	0.482	0.563	0.856	irs	62	0
TC03	0.586	0.589	0.994	irs	59	0
TC04	1	1	1	–	3	38
TC05	0.612	0.846	0.723	drs	23	0
TC06	0.523	0.634	0.824	irs	45	0
TC07	0.804	0.807	0.996	irs	26	0
TC08	0.536	0.703	0.764	irs	33	0
TC09	1	1	1	–	2	41
TC10	0.650	0.721	0.901	irs	32	0
BC01	0.719	0.741	0.971	drs	31	0
BC02	0.276	0.506	0.547	irs	71	0
BC03	0.496	0.682	0.727	irs	35	0
BC04	0.466	0.523	0.892	irs	68	0
BC05	0.284	0.795	0.357	irs	29	0
BC06	0.808	0.879	0.918	drs	20	0
BC07	0.611	1	0.611	drs	4	15
BC08	0.310	1	0.310	irs	1	43
BC09	0.821	1	0.821	drs	8	7
RC01	0.622	0.767	0.810	drs	30	0
RC02	0.521	0.679	0.768	drs	36	0
RC03	0.367	0.512	0.717	irs	69	0
RC04	0.466	0.567	0.822	irs	61	0
RC05	0.169	0.446	0.380	irs	74	0
RC06	0.125	0.287	0.435	irs	76	0
RC07	0.672	0.955	0.704	drs	16	0
RC08	0.577	0.674	0.856	drs	39	0
RC09	0.437	0.831	0.526	irs	25	0
RC10	0.314	0.593	0.530	irs	57	0
Minimum	0.125	0.287	0.310			
Median	0.483	0.674	0.726			
Mean	0.508	0.721	0.700			
Maximum	1	1	1			
Standard deviation	0.207	0.185	0.200			

OTE, overall technical efficiency; PTE, pure technical efficiency; SE, scale efficiency; irs, increasing return to scale; drs, decreasing return to scale.

a Peer refers to the number of other DMUs being benchmarked against it.

### 3.3. Improvement of efficiency score for ambulance services

Table 3 illustrates the output increments and input reduction that would allow the variable return to scale of the pure technical inefficient DMUs to achieve the 100% efficient level. There were 65 pure technical inefficient DMUs (hospitals and health clinics) in total. The mean increment of the number of patients transferred, the distance coverage, and the hours of usage were 14 (±22) patients, 189.94 (±370.28) km, and 19.78 (±71.89) hours, respectively (Table 3). To be 100% efficient, all the DMUs (100.00%) must reduce the cost of ambulance services as was projected (Table 4). The mean cost reduction was MYR 8,774.30 (±13,491.25). With the newly projected cost, 64.62% of the DMUs shall increase the number of patients transferred, 35.38% need to increase the distance coverage, and 27.69% need to increase the hours of usage (Table 4).

**Table 3:** Projected outputs and input for ambulance services for pure technical inefficient DMUs (*n* = 65).

**DMUs**	**Output/input**		**Actual**	**Projected**	**Difference**	**%**
**Hospitals**						
SH01	Output	Distance coverage (in km)	17,242.00	18,885.42	1,643.42	9.53
		Number of patients transferred	294	436	142	48.30
		Hours of usage (in hours)	859.87	859.87	0.00	0.00
	Input	Cost (in MYR)	144,911.47	83,039.65	−61,871.82	−42.70
JH01	Output	Distance coverage (in km)	16,466.25	16,466.25	0.00	0.00
		Number of patients transferred	287	287	0	0.00
		Hours of usage (in hours)	500.42	581.259	80.84	16.15
	Input	Cost (in MYR)	89,281.13	62,128.2	−27,152.93	−30.41
KH01	Output	Distance coverage (in km)	21,791.75	21,791.75	0.00	0.00
		Number of patients transferred	194	194	0	0.00
		Hours of usage (in hours)	642.17	642.17	0.00	0.00
	Input	Cost (in MYR)	95,394.12	81,497.16	−13,896.96	−14.57
TH01	Output	Distance coverage (in km)	15,255.75	15,255.75	0.00	0.00
		Number of patients transferred	129	207	78	60.47
		Hours of usage (in hours)	592.50	592.50	0.00	0.00
	Input	Cost (in MYR)	89,341.48	56,578.89	−32,762.59	−36.67
TH02	Output	Distance coverage (in km)	15,460.25	15,460.25	0.00	0.00
		Number of patients transferred	328	328	0	0.00
		Hours of usage (in hours)	374.56	670.077	295.52	78.90
	Input	Cost (in MYR)	128,225.04	62,267.62	−65,957.42	−51.44
BH01	Output	Distance coverage (in km)	15,887.25	15,887.25	0.00	0.00
		Number of patients transferred	476	476	0	0.00
		Hours of usage (in hours)	410.19	690.991	280.80	68.46
	Input	Cost (in MYR)	137,393.87	82,975.14	−54,418.73	−39.61
BH02	Output	Distance coverage (in km)	14,306.00	14,306.00	0.00	0.00
		Number of patients transferred	163	163	0	0.00
		Hours of usage (in hours)	322.53	351.233	28.70	8.90
	Input	Cost (in MYR)	80,917.70	44,264.92	−36,652.78	−45.30
RH01	Output	Distance coverage (in km)	19,082.50	19,082.50	0.00	0.00
		Number of patients transferred	57	102	45	78.95
		Hours of usage (in hours)	405.74	405.74	0.00	0.00
	Input	Cost (in MYR)	64,306.50	62,082.30	−2,224.20	−3.46
RH02	Output	Distance coverage (in km)	1,447.50	1,447.50	0.00	0.00
		Number of patients transferred	5	27	22	440.00
		Hours of usage (in hours)	50.44	50.44	0.00	0.00
	Input	Cost (in MYR)	16,241.04	4,832.12	−11,408.92	−70.25
RH04	Output	Distance coverage (in km)	19,937.00	19,937.00	0.00	0.00
		Number of patients transferred	539	539	0	0.00
		Hours of usage (in hours)	438.63	868.088	429.46	97.91
	Input	Cost (in MYR)	125,427.28	100,049.62	−25,377.66	−20.23
**Health clinics**						
SC01	Output	Distance coverage (in km)	2,784.25	3,388.21	603.96	21.69
		Number of patients transferred	80	104	24	30.00
		Hours of usage (in hours)	222.21	222.21	0.00	0.00
	Input	Cost (in MYR)	18,841.53	11,349.49	−7,492.04	−39.76
SC02	Output	Distance coverage (in km)	1,877.50	2,547.43	669.93	35.68
		Number of patients transferred	58	76	18	31.03
		Hours of usage (in hours)	161.96	161.96	0.00	0.00
	Input	Cost (in MYR)	9,233.35	9,042.43	−190.92	−2.07
SC03	Output	Distance coverage (in km)	455.50	500.58	45.08	9.90
		Number of patients transferred	39	39	0	0.00
		Hours of usage (in hours)	26.37	26.37	0.00	0.00
	Input	Cost (in MYR)	7,367.07	4,366.63	−3,000.44	−40.73
SC04	Output	Distance coverage (in km)	1,933.00	2,507.10	574.10	29.70
		Number of patients transferred	52	74	22	42.31
		Hours of usage (in hours)	159.07	159.07	0.00	0.00
	Input	Cost (in MYR)	9,822.69	8,931.77	−890.92	−9.07
SC05	Output	Distance coverage (in km)	689.25	1,500.30	811.05	117.67
		Number of patients transferred	24	24	0	0.00
		Hours of usage (in hours)	33.77	33.77	0.00	0.00
	Input	Cost (in MYR)	6,318.89	4,286.16	−2,032.73	−32.17
SC06	Output	Distance coverage (in km)	1,094.75	1,550.43	455.68	41.62
		Number of patients transferred	29	29	0	0.00
		Hours of usage (in hours)	54.44	54.44	0.00	0.00
	Input	Cost (in MYR)	6,274.02	4,991.47	−1,282.55	−20.44
SC07	Output	Distance coverage (in km)	4,059.50	4,871.48	811.98	20.00
		Number of patients transferred	120	154	34	28.33
		Hours of usage (in hours)	328.50	328.5	0.00	0.00
	Input	Cost (in MYR)	25,557.33	15,419.49	−10,137.84	−39.67
SC08	Output	Distance coverage (in km)	2,818.75	3,032.64	213.89	7.59
		Number of patients transferred	85	92	7	8.24
		Hours of usage (in hours)	196.73	196.73	0.00	0.00
	Input	Cost (in MYR)	10,761.49	10,373.82	−387.67	−3.60
SC10	Output	Distance coverage (in km)	516.75	1,045.18	528.43	102.26
		Number of patients transferred	17	25	8	47.06
		Hours of usage (in hours)	54.31	54.31	0.00	0.00
	Input	Cost (in MYR)	8,055.48	4,920.35	−3,135.13	−38.92
SC11	Output	Distance coverage (in km)	6,129.00	6,129	0.00	0.00
		Number of patients transferred	55	55	0	0.00
		Hours of usage (in hours)	175.63	175.63	0.00	0.00
	Input	Cost (in MYR)	21,293.30	14,276.09	−7,017.21	−32.96
SC12	Output	Distance coverage (in km)	4,073.25	4,073.25	0.00	0.00
		Number of patients transferred	122	122	0	0.00
		Hours of usage (in hours)	196.00	206.29	10.29	5.25
	Input	Cost (in MYR)	11,854.74	11,202.42	−652.32	−5.50
JC01	Output	Distance coverage (in km)	5,242.50	5,242.5	0.00	0.00
		Number of patients transferred	71	80	9	12.68
		Hours of usage (in hours)	201.40	201.4	0.00	0.00
	Input	Cost (in MYR)	18,731.48	12,627.65	−6,103.83	−32.59
JC02	Output	Distance coverage (in km)	4,806.50	4,806.5	0.00	0.00
		Number of patients transferred	37	51	14	37.84
		Hours of usage (in hours)	99.50	99.5	0.00	0.00
	Input	Cost (in MYR)	13,797.32	8,926.5	−4,870.82	−35.30
JC03	Output	Distance coverage (in km)	2,369.50	3,375.09	1,005.59	42.44
		Number of patients transferred	80	104	24	30.00
		Hours of usage (in hours)	221.27	221.27	0.00	0.00
	Input	Cost (in MYR)	24,537.16	11,313.49	−13,223.67	−53.89
JC04	Output	Distance coverage (in km)	3,144.75	3,144.75	0.00	0.00
		Number of patients transferred	41	64	23	56.10
		Hours of usage (in hours)	115.88	115.88	0.00	0.00
	Input	Cost (in MYR)	13,333.95	7,440.92	−5,893.03	−44.20
JC05	Output	Distance coverage (in km)	7,481.75	7,481.75	0.00	0.00
		Number of patients transferred	162	180	18	11.11
		Hours of usage (in hours)	392.14	392.14	0.00	0.00
	Input	Cost (in MYR)	29,342.45	24,412.26	−4,930.19	−16.80
JC06	Output	Distance coverage (in km)	1,339.25	2,862.797	1,523.55	113.76
		Number of patients transferred	57	57	0	0.00
		Hours of usage (in hours)	103.35	103.35	0.00	0.00
	Input	Cost (in MYR)	11,789.04	6,947.05	−4,841.99	−41.07
JC07	Output	Distance coverage (in km)	993.75	993.75	0.00	0.00
		Number of patients transferred	80	80	0	0.00
		Hours of usage (in hours)	41.73	43.42	1.69	4.05
	Input	Cost (in MYR)	8,427.73	5,638.24	−2,789.49	−33.10
JC08	Output	Distance coverage (in km)	3,641.25	3,641.25	0.00	0.00
		Number of patients transferred	51	66	15	29.41
		Hours of usage (in hours)	110.25	110.25	0.00	0.00
	Input	Cost (in MYR)	12,003.96	7,300.89	−4,703.07	−39.18
JC09	Output	Distance coverage (in km)	6,757.50	6,757.5	0.00	0.00
		Number of patients transferred	96	111	15	15.63
		Hours of usage (in hours)	264.25	264.25	0.00	0.00
	Input	Cost (in MYR)	29,083.05	18,296.09	−10,786.96	−37.09
JC10	Output	Distance coverage (in km)	2,312.00	2,312.00	0.00	0.00
		Number of patients transferred	51	64	13	25.49
		Hours of usage (in hours)	134.09	134.09	0.00	0.00
	Input	Cost (in MYR)	12,880.11	7,995.41	−4,884.70	−37.92
KC01	Output	Distance coverage (in km)	1,571.75	1,571.75	0.00	0.00
		Number of patients transferred	22	25	3	13.64
		Hours of usage (in hours)	43.38	43.38	0.00	0.00
	Input	Cost (in MYR)	7,318.69	4,591.05	−2,727.64	−37.27
KC02	Output	Distance coverage (in km)	931.75	931.75	0.00	0.00
		Number of patients transferred	63	63	0	0.00
		Hours of usage (in hours)	28.47	38.00	9.53	33.46
	Input	Cost (in MYR)	8,384.58	5,157.44	−3,227.14	−38.49
KC03	Output	Distance coverage (in km)	3,052.50	3,052.50	0.00	0.00
		Number of patients transferred	48	68	20	41.67
		Hours of usage (in hours)	128.19	128.19	0.00	0.00
	Input	Cost (in MYR)	14,457.13	7,877.60	−6,579.53	−45.51
KC04	Output	Distance coverage (in km)	1,023.00	1,023.00	0.00	0.00
		Number of patients transferred	17	19	2	11.76
		Hours of usage (in hours)	39.50	39.50	0.00	0.00
	Input	Cost (in MYR)	8,290.01	4,377.50	−3,912.51	−47.20
KC05	Output	Distance coverage (in km)	630.75	630.75	0.00	0.00
		Number of patients transferred	38	38	0	0.00
		Hours of usage (in hours)	25.80	27.60	1.80	6.97
	Input	Cost (in MYR)	8,347.56	4,381.98	−3,965.58	−47.51
KC06	Output	Distance coverage (in km)	2,013.25	2,595.57	582.32	28.92
		Number of patients transferred	73	77	4	5.48
		Hours of usage (in hours)	165.41	165.41	0.00	0.00
	Input	Cost (in MYR)	10,619.76	9,174.53	−1,445.23	−13.61
KC07	Output	Distance coverage (in km)	2,056.50	2,056.50	0.00	0.00
		Number of patients transferred	21	28	7	33.33
		Hours of usage (in hours)	34.05	41.357	7.31	21.46
	Input	Cost (in MYR)	4,763.16	4,580.97	−182.19	−3.82
KC08	Output	Distance coverage (in km)	702.00	758.97	56.97	8.12
		Number of patients transferred	10	15	5	50.00
		Hours of usage (in hours)	33.80	33.80	0.00	0.00
	Input	Cost (in MYR)	8,091.03	4,135.00	−3,956.03	−48.89
KC09	Output	Distance coverage (in km)	967.25	1,020.73	53.48	5.53
		Number of patients transferred	19	19	0	0.00
		Hours of usage (in hours)	38.28	38.28	0.00	0.00
	Input	Cost (in MYR)	6,762.81	4,332.72	−2,430.09	−35.93
KC10	Output	Distance coverage (in km)	1,336.75	1,336.75	0.00	0.00
		Number of patients transferred	7	19	12	171.43
		Hours of usage (in hours)	5.51	30.38	24.87	451.42
	Input	Cost (in MYR)	6,306.96	4,086.33	−2,220.63	−35.21
KC11	Output	Distance coverage (in km)	4,140.00	4,140.00	0.00	0.00
		Number of patients transferred	80	122	42	52.50
		Hours of usage (in hours)	254.58	254.58	0.00	0.00
	Input	Cost (in MYR)	13,493.89	12,628.41	−865.48	−6.41
TC02	Output	Distance coverage (in km)	2,873.50	2,873.50	0.00	0.00
		Number of patients transferred	21	42	21	100.00
		Hours of usage (in hours)	62.50	62.5	0.00	0.00
	Input	Cost (in MYR)	9,704.38	5,459.15	−4,245.23	−43.75
TC03	Output	Distance coverage (in km)	4,621.75	4,621.75	0.00	0.00
		Number of patients transferred	64	125	61	95.31
		Hours of usage (in hours)	253.88	253.88	0.00	0.00
	Input	Cost (in MYR)	21,486.72	12,666.18	−8,820.54	−41.05
TC05	Output	Distance coverage (in km)	6,821.00	6,821.00	0.00	0.00
		Number of patients transferred	45	52	7	15.56
		Hours of usage (in hours)	90.78	90.78	0.00	0.00
	Input	Cost (in MYR)	16,677.45	14,102.87	−2,574.58	−15.44
TC06	Output	Distance coverage (in km)	2,373.50	3,319.41	945.91	39.85
		Number of patients transferred	57	57	0	0.00
		Hours of usage (in hours)	93.56	93.56	0.00	0.00
	Input	Cost (in MYR)	10,485.65	6,650.12	−3,835.53	−36.58
TC07	Output	Distance coverage (in km)	4,819.25	4,819.25	0.00	0.00
		Number of patients transferred	132	142	10	7.58
		Hours of usage (in hours)	294.45	294.45	0.00	0.00
	Input	Cost (in MYR)	17,551.26	14,171.23	−3,380.03	−19.26
TC08	Output	Distance coverage (in km)	1,128.50	2,042.12	913.62	80.96
		Number of patients transferred	58	59	0	0.00
		Hours of usage (in hours)	125.75	125.75	0.00	0.00
	Input	Cost (in MYR)	10,896.42	7,655.90	−3,240.52	−29.74
TC10	Output	Distance coverage (in km)	3,159.75	3,159.75	0.00	0.00
		Number of patients transferred	64	78	14	21.88
		Hours of usage (in hours)	154.69	154.69	0.00	0.00
	Input	Cost (in MYR)	12,289.67	8,857.83	−3,431.84	−27.92
BC01	Output	Distance coverage (in km)	1,742.25	1,742.25	0.00	0.00
		Number of patients transferred	170	170	0	0.00
		Hours of usage (in hours)	140.10	146.755	6.66	4.75
	Input	Cost (in MYR)	14,325.54	10,609.79	−3,715.75	−25.94
BC02	Output	Distance coverage (in km)	944.75	1,086.21	141.46	14.97
		Number of patients transferred	15	26	11	73.33
		Hours of usage (in hours)	57.25	57.25	0.00	0.00
	Input	Cost (in MYR)	9,952.72	5,032.93	−4,919.79	−49.43
BC03	Output	Distance coverage (in km)	1,795.25	1,795.25	0.00	0.00
		Number of patients transferred	37	43	6	16.22
		Hours of usage (in hours)	88.54	88.54	0.00	0.00
	Input	Cost (in MYR)	9,184.73	6,266.86	−2,917.87	−31.77
BC04	Output	Distance coverage (in km)	3,042.25	3,042.25	0.00	0.00
		Number of patients transferred	75	85	10	13.33
		Hours of usage (in hours)	176.49	176.49	0.00	0.00
	Input	Cost (in MYR)	18,442.08	9,637.17	−8,804.91	−47.74
BC05	Output	Distance coverage (in km)	307.50	509.07	201.57	65.55
		Number of patients transferred	26	26	0	0.00
		Hours of usage (in hours)	3.37	22.87	19.50	578.61
	Input	Cost (in MYR)	5,054.99	4,017.19	−1,037.80	−20.53
BC06	Output	Distance coverage (in km)	4,363.50	4,363.50	0.00	0.00
		Number of patients transferred	45	53	8	17.78
		Hours of usage (in hours)	45.25	72.19	26.94	59.52
	Input	Cost (in MYR)	8,082.99	7,107.98	−975.01	−12.06
RC01	Output	Distance coverage (in km)	5,359.75	5,359.75	0.00	0.00
		Number of patients transferred	24	53	29	120.83
		Hours of usage (in hours)	63.00	77.67	14.67	23.29
	Input	Cost (in MYR)	12,893.69	9,895.87	−2,997.82	−23.25
RC02	Output	Distance coverage (in km)	6,282.50	6,282.50	0.00	0.00
		Number of patients transferred	33	46	13	39.39
		Hours of usage (in hours)	157.88	157.88	0.00	0.00
	Input	Cost (in MYR)	20,960.59	14,226.29	−6,734.30	−32.13
RC03	Output	Distance coverage (in km)	1,927.00	1,927.00	0.00	0.00
		Number of patients transferred	20	35	15	75.00
		Hours of usage (in hours)	63.88	63.88	0.00	0.00
	Input	Cost (in MYR)	10,527.85	5,385.11	−5,142.74	−48.85
RC04	Output	Distance coverage (in km)	2,302.75	2,439.84	137.09	5.95
		Number of patients transferred	53	72	19	35.85
		Hours of usage (in hours)	154.25	154.25	0.00	0.00
	Input	Cost (in MYR)	15,420.88	8,747.20	−6,673.68	−43.28
RC05	Output	Distance coverage (in km)	245.50	507.22	261.72	106.61
		Number of patients transferred	27	27	0	0.00
		Hours of usage (in hours)	18.00	23.125	5.13	28.47
	Input	Cost (in MYR)	9,075.02	4,043.68	−5,031.34	−55.44
RC06	Output	Distance coverage (in km)	684.25	811.44	127.19	18.59
		Number of patients transferred	13	17	4	30.77
		Hours of usage (in hours)	37.56	37.56	0.00	0.00
	Input	Cost (in MYR)	14,900.36	4,278.97	−10,621.39	−71.28
RC07	Output	Distance coverage (in km)	7,567.00	7,567.00	0.00	0.00
		Number of patients transferred	43	53	10	23.26
		Hours of usage (in hours)	71.69	89.822	18.13	25.29
	Input	Cost (in MYR)	16,838.60	16,072.60	−766.00	−4.55
RC08	Output	Distance coverage (in km)	5,669.00	5,669.00	0.00	0.00
		Number of patients transferred	49	61	12	24.49
		Hours of usage (in hours)	194.88	194.88	0.00	0.00
	Input	Cost (in MYR)	20,085.23	13,531.64	−6,553.59	−32.63
RC09	Output	Distance coverage (in km)	1,103.75	1,103.75	0.00	0.00
		Number of patients transferred	27	27	0	0.00
		Hours of usage (in hours)	25.69	30.018	4.33	16.85
	Input	Cost (in MYR)	5,098.00	4,238.16	−859.84	−16.87
RC10	Output	Distance coverage (in km)	1,117.50	1,421.22	303.72	27.18
		Number of patients transferred	23	23	0	0.00
		Hours of usage (in hours)	40.70	40.7	0.00	0.00
	Input	Cost (in MYR)	7,547.33	4,473.56	−3,073.77	−40.73
Total	Output	Distance coverage (in km)	310,073.75	322,685.46	12,611.71	–
		Number of patients transferred	5,398.00	6,275.00	876.00	–
		Hours of usage (in hours)	11,101.01	12,367.16	1,266.17	–
	Input	Cost (in MYR)	1,675,063.50	1,112,226.05	−562,837.45	–
Mean	Output	Distance coverage (in km)	4,827.80	5,017.73	189.94	–
		Number of patients transferred	84	98	14	–
		Hours of usage (in hours)	172.60	192.39	19.78	–
	Input	Cost (in MYR)	26,074.84	17,300.54	−8,774.30	–

**Table 4:** DMUs required improvement in outputs and input for ambulance services (*n* = 65).

**Output/input**		**Number of DMUs (%)**		

		**Require increment**	**Require reduction**	**Require no change**
Output	Distance coverage (in km)	42 (64.62)	–	23 (35.38)
	Number of patients transferred	23 (35.38)	–	42 (64.62)
	Hours of usage (in hours)	18 (27.69)	–	47 (72.23)
Input	Cost (in MYR)	–	65 (100.00)	0 (0.00)

### OTE, PTE, and SE of health facility and geographical area

Table 5 shows the mean and median of OTE, PTE, and SE by type of health facility. A Mann–Whitney *U*-test was conducted to compare the OTE, PTE, and SE between types of health facilities. There was a significant difference in OTE value for health clinics (median = 0.527) and Hospitals (median = 0.353; *U* = 1,026, *z* = 3.430, *p* = < 0.001). The magnitude of the differences was moderate (*r* = 0.39).

**Table 5:** OTE, PTE, and SE by type of health facility (*n* = 76).

	**Mean (SD)**		**Median (IQR)**		***p*-value**

	**Hospital**	**Clinic**	**Hospital**	**Clinic**	
OTE^a^	0.352 (0.090)	0.544 (0.211)	0.353 (0.138)	0.527 (0.281)	<0.001^***^
PTE^a^	0.747 (0.231)	0.715 (0.177)	0.747 (0.427)	0.672 (0.271)	0.511
SE^a^	0.485 (0.071)	0.749 (0.189)	0.486 (0.094)	0.810 (0.332)	<0.001^***^

*** *p* < 0.001.

a Mann–Whitney *U*-test.

OTE, overall technical efficiency; PTE, pure technical efficiency; SE, scale efficiency; SD, standard deviation. Values in bold are statistically significant.

There was also a significant difference in SE value for health clinics (median = 0.810) and hospitals (median = 0.486; *U* = 1,092, *z* = 4.315, *p* = < 0.001) with a moderate magnitude of differences (*r* = 0.49).

Nonetheless, there was no significant difference in PTE value for health clinics (median = 0.672) and hospitals (median = 0.747; *U* = 721, *z* = −0.658, *p* = 0.511).

Furthermore, under the health facility, the OTE of health clinics is influenced by SE rather than PTE because the efficiency of SE (mean = 0.749) is higher than PTE (mean = 0.715). This indicates that, although the clinics have been operating on a relatively optimal scale, their inefficiency is due to managerial factors. Therefore, the component of PTE constitutes the main factor in this analysis and warrants focus since the inefficiency of the OTE is contributed by PTE.

Meanwhile, the hospital efficiency is contributed by PTE (mean = 0.747) rather than SE (mean = 0.485). This suggests that hospitals are operating at an optimum level of efficiency on the managerial side of managing the resources but at the wrong scale.

The mean and median scores of OTE, PTE, and SE by health facility geographical areas are shown in Table 6. A Mann–Whitney *U*-test was conducted to compare the OTE, PTE, and SE between different health facility geographical areas. There was a significant difference in OTE value for health facilities located in rural (median = 0.527) and urban areas (median = 0.458; *U* = 593, *z* = 2.070, *p* = 0.038). The magnitude of the differences was small (*r* = 0.24).

**Table 6:** OTE, PTE, and SE by geographical area (*n* = 76).

	**Mean (SD)**		**Median (IQR)**		***p*-value**

	**Urban**	**Rural**	**Urban**	**Rural**	
OTE^a^	0.454 (0.173)	0.568 (0.229)	0.458 (0.246)	0.527 (0.415)	**0.038** ^*^
PTE^a^	0.683 (0.185)	0.763 (0.181)	0.618 (0.260)	0.744 (0.317)	0.055
SE^a^	0.672 (0.211)	0.732 (0.188)	0.720 (0.404)	0.811 (0.277)	0.131

* *p* < 0.05.

a Mann–Whitney *U*-test.

OTE, overall technical efficiency; PTE, pure technical efficiency; SE, scale efficiency; SD, standard deviation. Values in bold are statistically significant.

However, there was no significant difference in PTE value for health facilities located in rural (median = 0.744) and urban areas (median = 0.618; *U* = 608, *z* = 1.917, *p* = 0.555). Similarly, no significant difference was found in SE value for health facilities located in rural (median = 0.811) and urban areas (median = 0.720; *U* = 711, *z* = 1.508, *p* = 0.131).

Both rural and urban's OTE are contributed by PTE, suggesting that the inefficiency of OTE is contaminated by SE.

### Return to scale of health facility and geographical area

Table 7 depicts the relationship between Return to Scale categories by the type of health facility and geographical area for the inefficient DMUs. Of the total health clinics, 72.90% were categorized as having increasing return to scale, whereas 92.90% of the hospitals were categorized as decreasing return to scale. A chi-square test indicated a significant association between Return to Scale categories and type of facility, $X_{\left(1,\;\text{n}=73\right)}^{2}$ = 20.422, *p* < 0.001, *phi* = −0.529. This showed that health clinics were more likely to be categorized as increasing return to scale than hospitals. This means a small increase in the input to the health clinics would be more likely to result in a higher increase in the outputs.

**Table 7:** Return to scale categories by type of health facility and geographical area for variable return to scale (OTE) inefficient DMUs (*n* = 73).

	***n***	**Return to scale**		***P*-value^a^**

		**drs (%)**	**irs (%)**	
Type of health facility				**<0.001** ^***^
#x000A0;Health clinics	59	16 (27.10)	43 (72.90)	
#x000A0;Hospitals	14	13 (92.90)	1 (7.10)	
Geographical area				0.813
#x000A0;Rural	34	14 (41.20)	20 (58.80)	
#x000A0;Urban	39	15 (38.50)	24 (61.50)	

*** *p* < 0.001.

a Chi square test.

irs, increasing return to scale; drs, decreasing return to scale. Values in bold are statistically significant.

Notwithstanding the above, about 58.80 and 61.50% of facilities located in rural and urban areas, respectively, were categorized as increasing return to scale. However, a chi-square test showed no significant association between the Return to Scale categories and the geographical area of the facility, $X_{\left(1,\text{ \#x000A0;}\text{n}=73\right)}^{2}$ = 0.056, *p* = 0.813, *phi* = 0.028.

## Discussion

Overall, the results revealed that the OTE is contributed by PTE rather than SE because the level of PTE (72.1%) is higher than SE (70%). This indicates that, although the hospital and clinic were more managerially efficient in controlling costs and managing the resources, they were mainly operating at the wrong scale of operations. In total, there were eight health facilities, namely, SH02, JH02, KH02, RH03, TC01, BC07, BC08, and BC09 with CRS inefficient due to scale inefficiencies. Out of these facilities, four were hospitals and the remaining four were health clinics. These scale inefficiencies indicate the extent to which the input (or size) of the ambulance services can be further reduced (in fixed proportion) after being projected to the VRS in order to achieve the CRS efficient frontier (45, 46). In other words, those eight health facilities should be able to achieve the CRS efficient level for ambulance services by further decreasing their input.

Most studies on ambulance services report on the performance rather than the efficiency measure of ambulance services. These performance measures were based on the selected performance indicators such as the Ambulance Response Time (ART), patient outcomes (for example, cardiac arrest survival), and patients' transport time among others (4, 39, 47). The studies on the efficiency of ambulance services using DEA are limited. It is also inappropriate to compare with the results of available studies due to the different mix of inputs and outputs. For example, efficiency studies for public ambulance services in Kenya revealed a better score with a mean technical efficiency of 90.6%, while in India, the mean technical efficiency score for ambulance services was 76.8% (40, 41). These efficiency scores, however, were derived from two inputs and one output approach, of which the cost of ambulance service and consumables were selected as the inputs, while the output was kilometers covered, which are different from the mix of inputs and outputs in the current study.

This current study also revealed that the OTE and SE for ambulance services differ significantly between hospitals and health clinics. Clinics have a significantly higher OTE and SE for ambulance services compared to the hospitals. PTE on the other hand did not show any significant difference between hospitals and health clinics. Thus, the lower overall technical efficiency of ambulance services in the hospitals compared to the health clinics was mainly attributed to scale inefficiency (or the size of operation) rather than the pure management issues or inefficiencies (PTE). To achieve similar scale efficiency as the ambulance service in health clinics, the hospitals must reduce the inputs proportionately to the outputs achieved, hence, moving the projected VRS efficiency frontier to the CRS efficiency frontier (46). Health clinics were found to produce better output with their limited resources compared to the hospitals. While the gross distribution of ambulances was quite balanced between hospitals and clinics, detailed analysis revealed that each hospital had a much higher capacity in terms of the number of ambulances compared to the health clinics (12, 13). Thus, health clinics required a much lower cost and human resources to operate the ambulance services than the hospitals.

In this study, out of 73 CRS inefficient health facilities, 60.27% (44) were increasing return to scale and the remaining 39.73% (29) health facilities were decreasing return to scale. For the 44 health facilities which provide the ambulance services, a small increase of input, which is the cost, would increase the outputs, namely, distance coverage, number of patients transferred, and hours of usage in a greater percentage. Whereas, for the 29 health facilities, an increase in the input would only increase a lesser percentage of outputs. Health clinics were known to operate with fewer resources especially in terms of manpower compared to hospitals (48). Most of the time, the personnel were shared among various departments due to understaffing (49). In the provision of ambulance services, unlike the hospitals, there was no designated personnel for ambulance services in the health clinics. Subsequent analysis from this study also revealed that the health clinics were significantly more likely to be increasing return to scale compared to the hospitals. Hence, additional increment in the input (or resources) to the health clinics was more likely to result in a greater increment in the outputs, compared to the hospitals.

To the policymakers, this study would provide a rough guide for the prioritization of resources for ambulance services in MOH Malaysia through a benchmarking approach. With the limited budget and higher demand for healthcare services, proper distribution of resources is required to provide the optimum output for ambulance services. This study allows policymakers and immediate managers to take necessary action in improving efficiency by minimizing the input (or the cost) and maximizing the outputs. Many efficiency studies in the health sector have been conducted to improve the healthcare systems in terms of resource allocation and prioritization (50–52). Inefficient use of health resources could lead to lost opportunities for other economic sectors such as education, limit the potential health gains due to insufficient or suboptimal treatment, deny others of treatment, and also reduce social contribution to healthcare financing, especially in countries with social health financing schemes (53).

This study is one of its kind looking into the perspective of ambulance service efficiency in the healthcare facilities in the MOH, Malaysia. There were previous studies on healthcare services focusing on the overall hospital efficiency level, efficiency of maternal health services at the health clinics, and performance of ambulance or emergency medical systems, but no documented studies focusing on ambulance services and benchmarking them in both hospitals and health clinics (34, 54). This study also employed the DEA approach to measure efficiency. DEA has been widely used for measuring efficiency not only in the health sector but also in other sectors such as the military, financing, and others (33). The DEA approach also allows detailed analysis of the DMUs through a mathematical program, thus making it possible to decompose OTE into PTE and SE for a better understanding of the DMU efficiency scores.

Nevertheless, this study is not without limitations. Cost as the input for the study may not be the most appropriate use of indicator since it is aggregated data and need to be interpreted with caution. The cost of ambulance services in this study consists of personnel cost, ambulance value, maintenance cost, overhead cost, and others. Therefore, while projecting the reduction of the cost, all these components of cost in providing ambulance services must be taken into account. In the economic analysis of the health sector, the preferred outcome is the improvement of quality of life, usually in the form of Quality Adjusted Life Years (QALYs) or Disability Adjusted Life Years (DALYs) (55). However, this study employed the immediate output of the ambulance services rather than the QALYs or DALYs due to practical limitations. Using QALYs or DALYs as the outcome measure may change the results of the study. Besides that, the use of DEA for measuring technical efficiency also has its own limitations. One of the limitations is the inability to determine the statistical significance of the DEA weights. Another limitation is that DEA does not take into consideration the random error in the model. There might be some biases in presenting the inefficiencies since DEA takes random fluctuations as inefficiency (56). The conventional DEA also depends on entirely well-defined data for the production set. However, the real-world data may not be necessarily well-defined. Input and output data are sometimes fuzzy and random in the real world. In addition, the conventional DEA does not take into consideration the DM's preference structure in the analysis.

## Conclusion

In conclusion, the ambulance services provided in healthcare facilities by the MOH Malaysia operate at 72.1% PTE. Only 14.47% of the health facilities were found VRS-efficient in providing ambulance services. The technical inefficiencies in the provision of ambulance services were mostly due to managerial inefficiency and inappropriate size (or scale inefficiency). However, results suggested that for the hospitals, the inefficiencies were mostly due to scale inefficiency rather than true managerial that cause the technical efficiency. Proper planning by the managers or the policymakers must ensure that the ambulance services are provided in the most efficient manner so that the right allocation of resources would result in optimum outputs. Future studies should investigate the ambulance service performance using the regression analysis approach or the Stochastic Frontier Analysis (SFA) for a better understanding of the inefficiency score by considering the random error. Detailed analysis of the determinant factors of the efficiency level would also complement and add value to the current study. Future studies may also incorporate MOLP in solving the DEA problem of ambulance efficiency to provide better performance assessment by taking into account the decision-making (DM) preferences.

## Data availability statement

The original contributions presented in the study are included in the article/supplementary material, further inquiries can be directed to the corresponding author.

## Author contributions

NM: conceptualization, writing the original draft, and formal analysis. MB: methodology, investigation, data curation, writing, reviewing, editing, and validation. FA, MM, NZ, and AM: writing—review and editing and validation. FK: writing, reviewing, editing, validation, and supervision. All authors contributed to the article and approved the submitted version.
